# The Ancestry of Eastern Paraguay: A Typical South American Profile with a Unique Pattern of Admixture

**DOI:** 10.3390/genes12111788

**Published:** 2021-11-12

**Authors:** Filipa Simão, Julyana Ribeiro, Carlos Vullo, Laura Catelli, Verónica Gomes, Catarina Xavier, Gabriela Huber, Martin Bodner, Alfredo Quiroz, Ana Paula Ferreira, Elizeu F. Carvalho, Walther Parson, Leonor Gusmão

**Affiliations:** 1 DNA Diagnostic Laboratory, State University of Rio de Janeiro, Rio de Janeiro 20550-013, Brazil; f.simao@campus.fct.unl.pt (F.S.); julyanaribeiro@ymail.com (J.R.); apfa_bio@yahoo.com.br (A.P.F.); elizeufc@hotmail.com (E.F.C.); 2 DNA Forensic Laboratory, Argentinean Forensic Anthropology Team, Córdoba 14001, Argentina; cvullo@yahoo.com.ar (C.V.); marialauracatelli@gmail.com (L.C.); 3 Instituto de Investigação e Inovação em Saúde, Universidade do Porto, 4099-002 Porto, Portugal; vgomes@ipatimup.pt; 4 Institute of Pathology and Molecular Immunology, University of Porto (IPATIMUP), 4099-002 Porto, Portugal; 5 Institute of Legal Medicine, Medical University of Innsbruck, 6020 Innsbruck, Austria; catarina.gomes@i-med.ac.at (C.X.); gabriela.e.huber@i-med.ac.at (G.H.); martin.bodner@i-med.ac.at (M.B.); 6 Instituto de Previsión Social, Asunción 100153, Paraguay; alfredoquiroz1974@me.com; 7 Forensic Science Program, The Pennsylvania State University, State College, PA 16801, USA

**Keywords:** mtDNA, Y chromosome, Y-STRs, Y-SNPs, AIM-InDels, ancestry

## Abstract

Immigrants from diverse origins have arrived in Paraguay and produced important demographic changes in a territory initially inhabited by indigenous Guarani. Few studies have been performed to estimate the proportion of Native ancestry that is still preserved in Paraguay and the role of females and males in admixture processes. Therefore, 548 individuals from eastern Paraguay were genotyped for three marker sets: mtDNA, Y-SNPs and autosomal AIM-InDels. A genetic homogeneity was found between departments for each set of markers, supported by the demographic data collected, which showed that only 43% of the individuals have the same birthplace as their parents. The results show a sex-biased intermarriage, with higher maternal than paternal Native American ancestry. Within the native mtDNA lineages in Paraguay (87.2% of the total), most haplogroups have a broad distribution across the subcontinent, and only few are concentrated around the Paraná River basin. The frequency distribution of the European paternal lineages in Paraguay (92.2% of the total) showed a major contribution from the Iberian region. In addition to the remaining legacy of the colonial period, the joint analysis of the different types of markers included in this study revealed the impact of post-war migrations on the current genetic background of Paraguay.

## 1. Introduction

**Figure 1:**
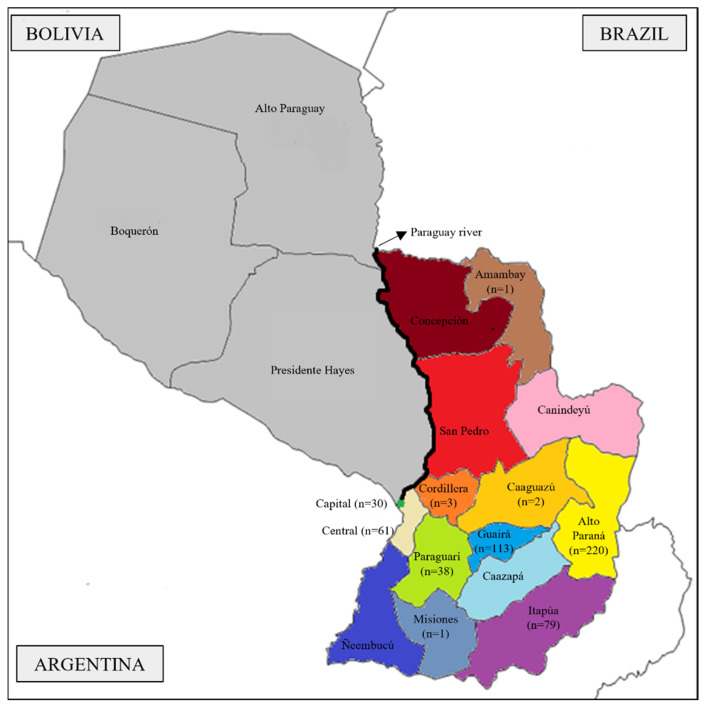
Map of Paraguay delimited by the surrounding countries of Argentina, Brazil, and Bolivia. Paraguay river (black line) separates the western departments (grey) from the eastern departments (colors). The information on the number of samples collected from each department, according to individuals’ living places, is indicated.

Paraguay is a landlocked country in the central region of South America. The country is divided by the Paraguay River into the eastern (or Paraneña) and the western (also known as Chaco) regions (Figure 1). Since the arrival of the first indigenous to the Paraguayan territory, the river acted as an important cultural barrier between individuals living in the two geographic regions. Indeed, the Guaranis that inhabited the western region lived a nomadic lifestyle, migrating in search of food, while the Guaranis living on the eastern part of the Paraguay River survived, mostly, from agriculture [1].

At the beginning of the 16th century, with the arrival of Spanish conquerors, political alliances were established with the Guarani natives living on the banks of Paraguay River. The admixture process began rapidly between Native American women and European men, and soon, the mestizos (a term used in Central and South America to describe people with admixed European and Native American ancestry) outnumbered the Spanish colonizers [2]. Although the coexistence between the indigenous and the Spanish began peacefully, in 1556, the “encomienda” system was implemented, where indigenous were enslaved and forced to work on the agriculture fields. Compared to other colonial empires at the time, the number of African slaves arriving to Paraguay was small [3,4], mainly because of the lack of large plantations and mines [5]. At the time of its independence in 1811, Paraguay had an admixed population resulting from intermarriage between Spanish colonizers, indigenous Guarani and few African slaves. 

The War of Triple Alliance, fought from 1864 to 1870, between Argentina, Brazil, and Uruguay against Paraguay, ended up in the demographic reduction of more than 50% of the Paraguayan population. Afterwards, immigration was encouraged by political measures. Contrary to expectations, the strategies did not create conditions to capture the flux of immigrants and only intensified the emigration movements. However, from the beginning of the 20th century, the arrival of foreigners in Paraguay began to be significant, without outnumbering the exit of Paraguayans from the country [6]. In this way, Germans, Ukrainians, Slavs and Japanese arrived in south and central east of the eastern region. Furthermore, immigrants from Russia, Germany, Canada, USA and Mexico moved to the Chaco region and afterwards to the north and central areas of the eastern region. Without having a specific territorial fixation, Spanish and Italians dispersed through the country. From the 1970s, the immigration of Brazilians began to be predominant after the opening of the agricultural border with Paraguay [6].

Due to its geographic isolation together with its poor economic conditions (lack of precious metals and subsistence mainly from agriculture), Paraguay remained isolated from the surrounding countries for decades [3,7]. Currently, the country is organized in 17 different departments (Figure 1). The western region harbors only 2.5% of the population while covering more than 50% of the territory. The remaining population lives in the eastern region comprising 14 departments and the capital city Asunción (Figure 1). 

As a reflection of the Paraguayan history of the last centuries, the genetic makeup of the country should reveal a different genetic history from those portrayed for other South American populations. Nonetheless, to date, no extensive knowledge about the genetic diversity of Paraguay and its stratification has been developed, and only little research is available for a limited number of markers. Previous studies for both autosomal and Y-chromosomal STRs (Y-STRs) reported allele/haplotype frequencies for eastern Paraguay [8,9]. With these markers, no statistically significant differentiation was detected among departments in the eastern Paraguay region. However, because these STRs were selected based on their forensic relevance, with high intrapopulation and low among population variance, they are not the most appropriate for evaluating population substructure or disclosing admixture patterns. Markers with low mutation rates are less prone to recurrent mutation and more likely to show geographic specificity. Thus, to evaluate biparental ancestry proportions or to determine the geographic origin of Y-lineages, biallelic polymorphisms are more often used than STRs.

Previous studies on mitochondrial DNA (mtDNA) were also performed for populations in the eastern region of Paraguay [10,11]. The results showed a predominant Native American maternal ancestry. Based on mitogenome data analysis, a multiple ethnic input for the maternal Native American lineages was hypothesized [11].

With the aim of furthering our knowledge of the impact of the different migratory movements in the current genetic background of Paraguay, an integrated analysis of marker sets with different transmission patterns was performed in this study. The sample size previously analyzed for mtDNA was enlarged [10], and this allowed for a more detailed tracking of the ethno-linguistic/geographic origin of the maternal lineages in Paraguay, in addition to the previous estimates on the ancestry from different continents. Moreover, specific Y chromosome SNPs (Y-SNPs) were genotyped for the samples previously studied for Y-STRs [9]. Although the results of the Y-STRs have shown a proximity between Paraguay and European populations, the exact proportion of European heritage is only possible with the classification of haplogroups based on Y-SNPs. For the same samples, a set of 46 ancestry informative indels (AIM-InDels), located in autosomes, was also analyzed. Information on mtDNA, Y-SNPs and AIM-InDels was used to estimate differential maternal and paternal contributions and their variation within and across geographic regions more accurately than in previous studies [9,10]. Furthermore, the data obtained were used to track the origin of maternal and paternal lineages in more detail and to determine how migration patterns shaped the country’s genetic makeup. 

## 2. Materials and Methods

### 2.1. Sampling

A total of 548 blood samples from unrelated individuals (8 females and 540 males) living in the eastern region of Paraguay were collected with informed consent. Detailed information on the number of samples from each department is depicted in Figure 1. The project and the informed consent were approved by the Ethics Committee in Clinical Research of the Institute of Social Security, Asuncion, Paraguay, and the ethical principles of the Helsinki Declaration of the World Medical Association [12] were followed.

DNA was extracted with Chelex resin using a conventional protocol described by Walsh et al. [13].

### 2.2. Genotyping Methods

In this study, 522 samples were genotyped for 46 AIM-InDels (see results in Supplementary Table S1) in a single PCR multiplex followed by Capillary electrophoresis, using the method described by Pereira et al. [14]. 

A total of 61 Y-SNPs were genotyped in 463 individuals. The Y-SNPs were selected to discriminate the most frequent haplogroups in African, Native American, European, and Asian populations. Y-STR haplotypes previously reported for these samples [9] were used for haplogroup prediction on Y-DNA Haplogroup Predictor NevGen [15]. This way, it was possible to direct genotyping and avoid extra typing. The Y-SNPs were grouped hierarchically into seven multiplexes, as described in Supplementary Figure S1 [16,17,18,19]. Three new PCR/SNaPshot multiplexes were designed to include Y-SNPs inside European and Asian haplogroups, as described in Supplementary Information S1.

In this study, 100 samples were genotyped for the mtDNA control region (CR) and 18 for the full mitogenome. Apart from these 118 samples, the full set of 537 mtDNA haplotypes used in this study comprised previously reported data on 299 CR haplotypes [10], as well as 120 mitogenomes included in Simão et al. [11] and in Strobl et al. [20] (sample codes LS177-LS187, LS504-507). 

Amplification, sequencing and analysis were performed according to the protocols described in Simão et al. [11,21]. Haplogroups were determined on EMPOP, based on Phylotree, build 17 [22]. For statistical assessments, the 537 haplotypes (399 control region and 138 mitogenome) were framed between positions 16,024 and 576, and the following indel positions were disregard: 16193.xC, 309.xC, 315.xC, 523–524del, 524.xC and 573.xC. The samples were submitted to EMPOP and are available under the accession numbers EMP00728 and EMP00835. Haplotypes and corresponding haplogroups of the 118 samples sequenced in this study, as well as the 299 included in Simão et al. [10], are detailed in Supplementary Table S2.

### 2.3. Statistical Analysis

For the AIM-InDels, the apportionment of each continental contribution was estimated using the software STRUCTURE v2.3.3 [23] with 200,000 burn-in steps followed by 200,000 Markov Chain Monte Carlo (MCMC) iterations. A tri-hybrid contribution from Native Americans, Europeans and Africans was assumed (K = 3) (data from [14,24,25,26]). Analysis was carried out using the “Use Population Information” option, and allele frequencies were correlated and updated using only individuals with POPFLAG = 1. A gradient map based on the proportions of European ancestry in admixed South American populations [14,24,25,27,28,29,30,31,32,33,34,35,36,37,38,39,40,41,42,43] was designed in the Surfer software v.16.0.330 (Golden Software, LLC, Golden, CO, USA), using the Kriging method for interpolating frequency values.

The Arlequin software [44] was used to evaluate inter- and intra-population diversities by mean of AMOVA and F_ST_ genetic distances. For mtDNA and Y-SNP haplogroups, AMOVA and pairwise F_ST_s were calculated using conventional F-statistics [45]. For mtDNA haplotype sequences, calculations were based on pairwise differences. The same software was used to calculate haplotype and haplogroup frequencies. To assist in visualizing the pairwise genetic distances obtained, multidimensional scaling (MDS) plots were constructed using the Statistica Software v. 14.0.0.15 (TIBCO Software Inc.).

To investigate the origin of the current paternal European background of Paraguay, comparisons were performed using Y-SNP data pooled from the literature by means of pairwise F_ST_s (as described above) and principal component analysis (PCA) using Statistica software v. 14.0.0.15 (TIBCO Software Inc.). Data from several European populations were collected [46,47,48,49,50,51,52,53], representing different geographic regions. In these analyses, the non-European haplogroups in the sample from Paraguay were excluded. Pairwise F_ST_ calculations and PCA were also performed for sub-lineages inside the R1b-M269 haplogroup. These analyses were based on frequency distributions of the following haplogroups: R1b-M269, R1b-L23, R1b-U106, R1b-S116, R1b-M529 and R1b-U152. European data were extracted from Adams et al. [46], Boattini et al. [48], Myres et al. [54] and Rebala et al. [52]. Existing data on Brazilian populations were also included for comparison [18].

For mtDNA, a comparison between the subsets of native mtDNA haplotypes in South American countries was performed using population data obtained from [21,34,55,56,57,58,59,60,61,62,63] (see detailed information in Supplementary Table S3). Gradient maps based on mtDNA haplogroup frequencies were designed in Surfer software v.16.0.330 (Golden Software, LLC), using the Kriging method for interpolating frequency values. To investigate associations between haplotypes and linguistic affiliation, and thus scrutinize the native component in the country, we searched for shared haplotypes among Paraguay and Native South American populations [26,62,63,64,65,66,67,68,69] (see details in Supplementary Table S4).

Proportions of maternal and paternal ancestries in admixed South American populations were retrieved from the literature [21,34,56,57,59,61,63,70,71,72,73,74,75,76,77,78,79,80,81,82,83,84,85,86,87,88,89] and represented in a bar chart, together with those obtained for Paraguay.

## 3. Results and Discussion

### 3.1. Demography and Genetic Structure of Eastern Paraguay

The sampling strategy of this study was designed to obtain a wide and detailed representation of each department in eastern Paraguay in order to evaluate population substructure. However, based on the demographic information collected from all individuals, a high mobility between departments during the last two generations was uncovered. In fact, 37% of the individuals did not reside in the department where they born. The same trend was observed when comparing individuals’ residence and parents’ birthplace. Differences were found in 47% of the cases when compared to the mother, and 62% when compared to the father’s birthplace (Supplementary Figure S2). 

When an AMOVA analysis was performed, low F_ST_ values (below 0.01) were obtained for the three marker sets after grouping the samples according to individuals’ place of residence or parents’ birthplace (Supplementary Table S5). Therefore, due to the high mobility detected between departments and the genetic homogeneity found among them, all samples were grouped as eastern Paraguay for further analyses. This genetic homogeneity across eastern Paraguay is in accordance with previous results from autosomal and Y-chromosomal STRs [8,9]. 

### 3.2. Continental Ancestry of Eastern Paraguay

**Figure 2:**
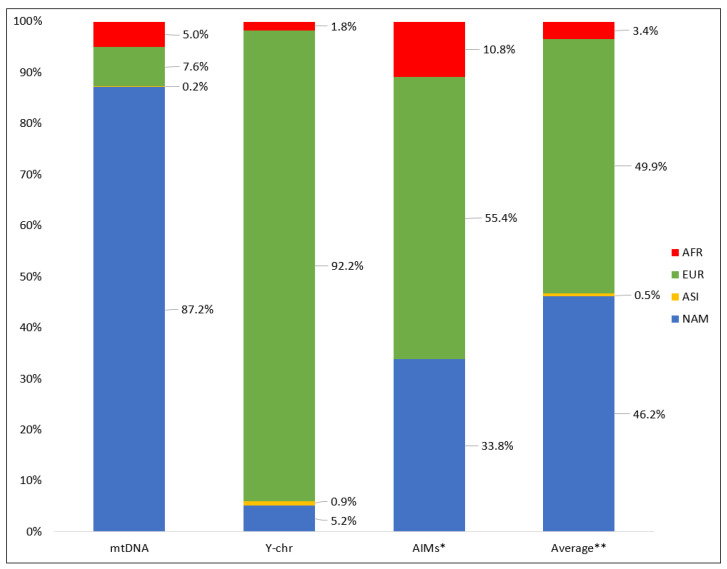
Ancestry proportions obtained with mtDNA (*n* = 537), Y chromosome (*n* = 463), 46 AIM-InDels (*n* = 422), and average of mtDNA and Y chromosome. AFR—African; EUR—European; ASI—Asian; NAM—Native American. * Ancestry estimates calculated for k=3. ** Average of mtDNA and Y chromosome ancestries.

The maternal genetic background of eastern Paraguay was predominantly of Native American ancestry, which is in agreement with the results obtained for a subset of 299 out of the 537 samples from this study [10]. The remaining samples belonged to African (5%) and European haplogroups (7.6%) (Figure 2). One sample was classified as A + 152 + 16362 + 200, a haplogroup of Asian origin. In this case, both parents were born in South Korea, the country where this haplogroup is frequent [90]. 

In contrast, Y-SNP data showed a predominance of European haplogroups. Samples with African, Native American, and Asian paternal ancestry were detected at low rates (Figure 2). 

**Figure 3:**
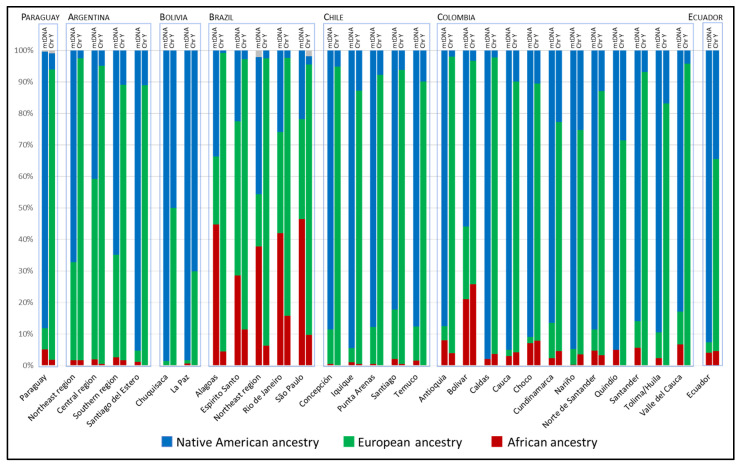
Maternal and paternal ancestry proportions in admixed South American populations. AFR—African; EUR—European; NAM—Native American.

Analyzing together the results obtained for uniparental lineages, a biased mating between European males and Native American females, typical of admixed populations from South America, can be observed. Nevertheless, the patterns of asymmetry are different across the subcontinent (Figure 3). Eastern Paraguay is among the populations with the greatest asymmetry between maternal and paternal lineages ancestry. A similar pattern can be seen in populations from Chile and Santiago del Estero (Argentina), and from some regions of Colombia. On average, the populations from Argentina show higher proportions of European maternal ancestry. In contrast with all other populations, Bolivia, and to lesser extent Ecuador, show a greater preservation of the native paternal component. Brazil differs from the other countries due to the high maternal contribution of African origin.

The genetic background of Paraguay was further evaluated with autosomal data obtained for 46 AIM-InDels. This multiplex contains informative markers for African, European, East Asian, and Native American ancestries. Nonetheless, a four-group analysis in South American admixed populations must be carefully considered, since it was previously demonstrated that Asian and Native American contributions cannot be fully resolved [14]. Namely, in the absence of Asian ancestry, an analysis including this as reference population will show a contribution withdrawn from the Native American and European ancestries. Therefore, considering the extremely low Asian input detected with the uniparental markers, the ancestry profile of eastern Paraguay was estimated considering only Africa, Native American and Europe as reference groups. The ancestry proportions obtained were as follows: 55.4% European, 33.8% Native American and 10.8% African (Figure 2). These values were different from those obtained by averaging lineage markers ancestry (Figure 2). The observed Native American autosomal ancestry was lower than expected. In contrast, the African and European contributions were higher than the average from mtDNA and Y chromosomes. This can be explained by the recent post-war influx of migrants. The European arrival to Paraguay in the last century, mainly of males, led to an increase in the European autosomal background, simultaneously with a decrease in the Native American one, but maintaining the maternal native component. 

The low African ancestry uncovered in this study was concordant with historical data that registered the arrival of a restricted number of Africans to Paraguay during the transatlantic slave trade when compared to the surrounding regions. Moreover, we cannot exclude that the observed African heritage resulted in part from Brazilian immigration, which was responsible for one of the major recent influxes in the country [6]. In fact, Pena et al. [91] detected a genomic proportion of African ancestry of around 10% in populations from South Brazil, which is geographically close to Paraguay. 

Immigration from Asia to eastern Paraguay took place since the 20th century [6], namely from China, Korea and Japan. Although low, it was possible to detect signs of these migrations in both paternal and maternal gene pools. Most individuals carrying Asian Y chromosome haplogroups had non-Asian mtDNA lineages, except one individual with both parents born in South Korea. These results showed that, after immigration, admixture events took place rather than an isolation of the Asian people.

### 3.3. Native American Maternal Ancestry

The high Native American ancestry found in eastern Paraguay is in agreement with the reported in several admixed populations from South America [34,55,61]. Haplogroups A, B, C and D comprise most of the native haplotypes in South American populations. The frequency of these lineages varies among populations, and some may be absent in certain regions. 

**Figure 4:**
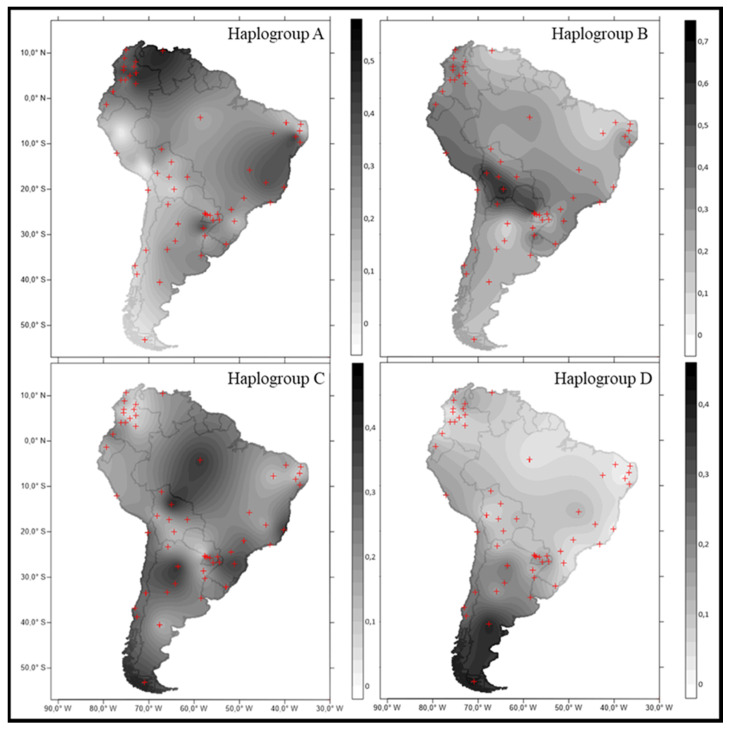
Frequency distributions of the native macrohaplogroups A, B, C and D in South American admixed populations. Latitude and longitude are represented at the left and bottom sides, respectively. Haplogroup frequencies are represented in grey scale according to the legend in the right side of the box. To facilitate interpretation, frequencies are scaled differently for each haplogroup. Red dots represent the location of population used (see Supplementary Table S3).

In admixed populations from South America, haplogroup A reaches the highest frequency around the Caribbean Sea coast (Venezuela and Colombia) (Figure 4). High frequencies are also observed on eastern South America, and geneflow southward along the Atlantic coast has been suggested as responsible for the presence of this haplogroup in Brazil and Argentina [92]. In eastern Paraguay, haplogroup A showed an intermediate frequency between the Andean region and the Atlantic coast, with values spanning from 15% to 26% among Paraguayan departments.

Haplogroup B has a high frequency along the Andes Mountain range (from Peru to North Argentina), reaching values above 50% in several populations from Peru, Bolivia and North Argentina (Figure 4). This lineage was detected in the sample from Asunción, at a rate of 63%, similar to the pattern observed along the cordillera (Figure 4). The remaining Paraguayan departments showed lower haplogroup B frequencies. Overall, this haplogroup accounted for 31% of the haplotypes observed in eastern Paraguay. 

Haplogroup C shows a wide distribution in the subcontinent, with frequency peaks in several regions, such as Bolivia, Brazil and Argentina (Figure 4). The frequency of this haplogroup in Paraguay (29%) was similar to that found in the southern region of Brazil [93]. 

In South America, haplogroup D presents an increasing gradient towards the south, reaching around 40% frequency in southern regions of Argentina and Chile (Figure 4). This haplogroup represented 17% of the haplotypes in eastern Paraguay, standing in accordance with the gradient pattern.

In summary, the analysis based on the distribution of haplogroups throughout South America did not allow for tracing of the origin of each native lineage in eastern Paraguay. This limitation was also found in the analysis of mitogenomes from the Alto Paraná, attributed to a lack of data from South American populations with the same level of resolution [11].

A comparison between the subsets of native mtDNA haplotypes in South American countries was performed. The MDS plot of pairwise F_ST_ genetic distances (Supplementary Figure S3) showed that Paraguay was closer to Brazilian populations than to populations from other countries. Before the European settlement in South America, the Tupi–Guarani natives inhabited a vast territory, from Brazilian northeast coast to Paraguay [94]. Thus, the similarity among the Brazilian and Paraguayan native pools might suggest a common background prior to the colonization period. However, although Tupi–Guarani must have been the major contributor to the current genetic background of Paraguay, the contribution of other ethnic/linguistic families cannot be excluded.

### 3.4. Phylogeographic Reconstruction of Maternal Native Lineages

Due to the absence of a clear pattern on macro-haplogroups distribution across South America, a phylogeographic approach was used to unveil geographic or linguistic affiliations of the native maternal lineages in Paraguay. Samples belonging to each of the native lineages found in Paraguay were retrieved from the literature (see references in Supplementary Table S3). The geographic origin and incidence of each haplogroup are illustrated in Supplementary Figures S4–S7. Most sublineages were previously reported in several regions, showing a wide distribution across the subcontinent. Nonetheless, exceptions were observed in all macrohaplogroups, and some lineages showed a narrow geographic incidence around Paraguay and its neighboring countries. Inside haplogroup A, this is true for the sublineages A2ah and A2aa (Supplementary Figure S4). Furthermore, the B2c2a, B2i1 and B2i2a1 lineages were also restricted to Paraguay and surrounding regions (Supplementary Figure S5). The C1d1d branch was confined to eastern Paraguay, Brazil and Argentina (Supplementary Figure S6). Inside haplogroup D, D1j1a2 was only found in the Argentinian sample set (Supplementary Figure S7). 

Although some lineages seem to be restricted to a region around the Paraná River basin, most native haplogroups showed a broad distribution, making its geographic origin unclear.

To scrutinize the native component in the country, shared haplotypes were searched among Paraguay and Native South American populations. For some haplotypes, matches were limited to a specific native group. For instance, four haplotypes inside haplogroups A2+64 (*n* = 1), B2b3a (*n* = 2) and B2i1(*n* = 1) were traced to Jê groups living in Central-West and North Brazil [67]. Moreover, three haplotypes inside haplogroups A2+64, B2b3a and D1 were previously reported in Mataco–Guaycurú natives from Argentina [66]. Two out of 11 C1b8 samples have the same haplotype as samples from Tupi–Guarani natives in Brazil [67]. One A2+64 and one B2h haplotypes were previously reported in Quechuas from Peru and Ecuador [69], respectively. One C1 haplotype was previously described only in two samples from a Peruvian native population speaking Jivaro [69]. Few D haplotypes were found in native communities from northwestern Amazonia [68]. 

Despite the expected genetic proximity with the neighboring countries, some native lineages previously reported in non-Guarani natives from northern South America were found in Paraguay. Therefore, the presence in Paraguay of lineages from different sources in addition to Tupi–Guarani cannot be discarded. Nonetheless, an association between genetics and linguistics or ethnicity can be misleading due to genetic flow that has been reported among native groups with different linguistic and/or ethnic backgrounds [68,95].

### 3.5. European Paternal Ancestry

Previous results based on Y-STRs showed a high genetic proximity between eastern Paraguay and South American populations with high paternal European ancestry [9]. The high European paternal ancestry was confirmed by the Y-SNP results obtained in the present study. The majority of Y-haplogroups (92.15%) in eastern Paraguay have a European origin, and just a small percentage of Native American (5.18%), African (1.80%) and Asian (0.86%) haplogroups were found in our sample (Supplementary Table S6).

**Figure 5:**
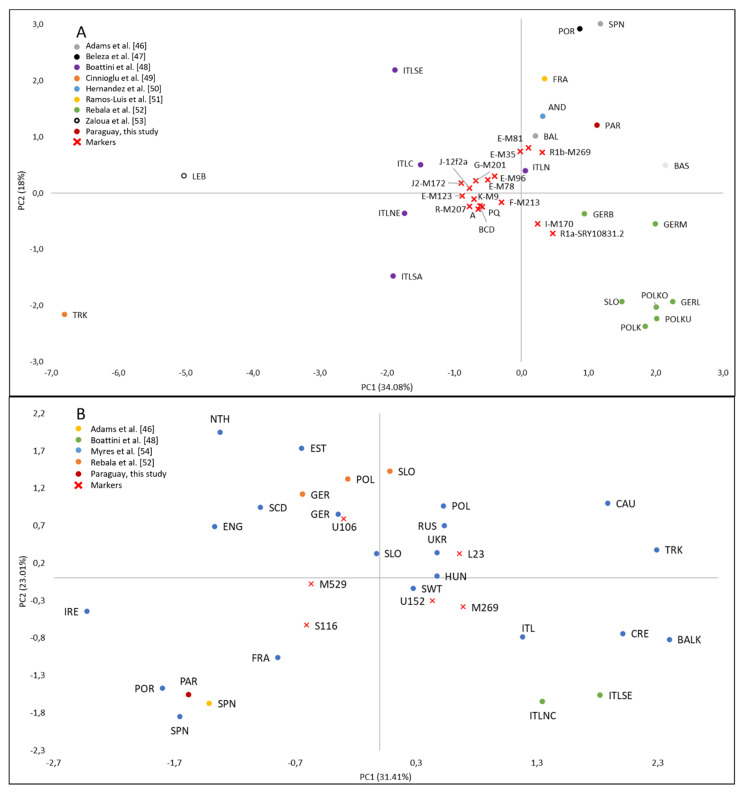
Principal component analysis of (**A**) European haplogroup frequencies in Paraguay and in European populations; (**B**) R1b-L23, R1b-M269, R1b-U106, R1b-S116, R1b-U152 and R1b-M529 haplogroup frequencies in Paraguay and in European populations. Legends: AND—Andalusia; BAL—Balearic; BALK—Balkans BAS—Basque; CAU—Caucasus; CRE—Crete; LEB—Lebanon; ENG—England; EST—Estonia; FRA—France; GER—Germany; GERB—Germany (Bavaria); GERL—Germany (Lusatia); GERM—Germany (Mecklenburg); HUN—Hungary; IRE—Ireland; ITL—Italy; ITLC—Italy (central); ITLSA—Italy (Sardinia); ITLSE—Italy (southeastern); ITLNC—Italy (north/central); ITLNE—Italy (northeastern); ITLN—Italy (northwestern); NTH—Netherlands; PAR—Paraguay; POL—Poland; POLK—Poland Kaszuby; POLKO—Poland Kociewie; POLKU—Poland Kurpe; POR—Portugal; RUS—Russia; SCD—Scandinavia; SLO—Slovakia; SPN—Spain; SWT—Switzerland; TRK—Turkey.

The MDS plot of the pairwise F_ST_ genetic distances based on haplogroup frequencies showed a proximity between samples from Paraguay and Iberia, France and northwestern Italy (Supplementary Figure S8). A similar result can be seen in the PCA (Figure 5A), showing a main contribution from Western Europe to Paraguay, supported by the high frequency of haplogroups inside R1b-M269 and the presence of the northwestern African haplogroup E1b1b-M81 [96]. The low pairwise F_ST_ values between Paraguay and Iberian samples (F_ST_ ≤ 0.0014; *p*-values ≥ 0.141) reveal no significant genetic drift in the European Y chromosome gene pool of Paraguay.

To further investigate the European substrate of Paraguay, we also compared sub-lineages inside the R1b-M269, the most frequent paragroup in our sample. In both the MDS representation of pairwise F_ST_ genetic distances (Supplementary Figure S9) and in the PCA (Figure 5B), Paraguay groups with Iberian samples due to the high frequency of haplogroup R1b-S116.

A new PCA was performed including samples from five Brazilian geographical regions [18] (the only South American data available for the same sublineages inside R1b-M269 that were included in the present study). The results show a high proximity between Paraguay and Brazilian populations (Supplementary Figure S10), with an overlap between the samples from Paraguay and the Southeast region of Brazil.

Combining our results and historical information available, it could be suggested that Spain may have been the major contributor to the current European paternal gene pool of Paraguay. However, it is worth noting that the studied markers do not allow differentiating Portugal and Spain, and the recent immigration from Brazil may have also carried Y chromosomes lineages from Portugal.

When analyzing the information obtained from mtDNA and Y chromosome together with autosomal genomes, some general trends can be observed. A lack of substructure is supported by all markers that show a homogeneity among the different departments, pointing to a high geneflow inside the country. Even so, the uniparental markers display different continental origins. A preservation of the ancient maternal background contrasts with the almost complete replacement of the native paternal lineages after the European arrival. A higher European contribution to the autosomal gene pool was found when compared to the expected based on lineage markers. This difference can be explained by several influxes of European males (rather than a single initial sex-biased admixture event) during colonization [74] and by postcolonial male-mediated migrations from countries with higher levels of autosomal and Y-chromosomal European ancestry. In fact, demographic data point to a significant migration to Paraguay from the neighboring countries of Argentina and Brazil after the War of Triple Alliance. As depicted in Supplementary Figure S11, Argentina and South Brazil present a higher proportion of autosomal European ancestry than Paraguay.

Due to the absence of recombination, the mtDNA and Y chromosome markers allow us to trace lineages’ history, even after admixture events. Paraguay mtDNA gene pool is almost exclusively of Native American ancestry, therefore harboring ancestral genetic footprints prior to the colonization period. No signs of isolation in relation to women who lived in the eastern region of Paraguay prior to the arrival of the Europeans were detected in this study. When attempting to determine the roots of the native mtDNA haplotypes found in Paraguay, affinities with both distant and neighboring populations were observed. It remains to be clarified whether the affinities found with distant populations represent (i) the footprints of the migratory routes of the first settlers arriving in the territory; (ii) genetic flow between native groups; (iii) or more recent migrations. It was not possible to carry out a comparative analysis based on the Y chromosome data, as the native inheritance of paternal origin was drastically erased. The low number of native paternal lineages detected in eastern Paraguay, together with the low resolution of South American native haplogroups, did not allow us to investigate their origin and dispersal routes. This analysis was also hampered by the low proportion of native lineages in neighboring populations, with little data available for comparison. However, from the Y-haplogroups of European origin found, it was possible to infer recent migrations, showing a high proximity to the Iberian populations. This result shows that, despite more recent arrivals of foreigners from other regions in Europe and Asia, the Iberian influence in South America during colonial times remains imprinted in the paternal gene pool of Paraguay. A genetic proximity was also found between Paraguay and Brazil, both for native maternal and European paternal lineages, supporting a continuous gene flow, beyond borders.

## 4. Conclusions

A sex-biased intercontinental admixture has been recurrently described for American populations and attributed to the admixture patterns during the European colonial period (e.g., [34,91]). The ancestry results obtained in this study showed the same trend in eastern Paraguay, with high Native American maternal and European paternal heritages. Despite this, it is possible to observe different patterns of asymmetry across the subcontinent, with eastern Paraguay being among the South American countries with the greatest asymmetry between maternal and paternal lineages ancestry. 

The South American native populations exhibit complex ancestry patterns due to genetic drift caused by bottleneck and/or founding effects [26,68,97,98]. For this reason, possible interactions between Native American populations can only be inferred from key lineages, whose description is invaluable for population genetics and forensics. However, it is difficult to perform geographic or ethnic/linguistic predictions based solely on mtDNA haplogroup diversity in native groups. Furthermore, among native groups, an intensive gene flow was reported between different language backgrounds, as well as language transmission without significant geneflow [68], further masking a correlation between linguistic and genetics. Nonetheless, the detection of mtDNA haplotypes restricted to dispersed native groups points to a multi-ethnolinguistic origin of the mtDNA gene pool of Paraguay.

The study of paternal lineages showed a European gene pool very similar to the one found in Iberian populations, with no significant differences between haplogroup frequencies, pointing to the absence of important genetic drift events. Reconciling our results with the historical reports, it is possible to conclude that Spain was the major source of the current European paternal ancestry in Paraguay.

## Data Availability

The data presented in this study are available in the Supplementary Materials.

## Supplementary Materials

The following are available online at https://www.mdpi.com/article/10.3390/genes12111788/s1, Figure S1: Phylogenetic tree of the Y-SNPs analyzed in this study, Figure S2: Distribution of the samples according to: (a) individual’s living place; (b) individual’s birthplace; (c) father’s birthplace; (d) mother’s birthplace, Figure S3: Multidimensional scaling (MDS) plot based on F_ST_ genetic distances of native mtDNA haplogroup frequencies among admixed populations from Argentina, Bolivia, Brazil, Chile, Colombia, Ecuador, Peru, Uruguay and Venezuela, Figure S4: Geographic origin and incidence in South America of the A lineages found in Paraguay, Figure S5: Geographic origin and incidence in South America of the B lineages found in Paraguay, Figure S6: Geographic origin and incidence in South America of the C lineages found in Paraguay, Figure S7: Geographic origin and incidence in South America of the D lineages found in Paraguay, Figure S8: MDS plot of the pairwise F_ST_ genetic distances between European Y chromosome haplogroup frequencies in Paraguay and in European populations that have potentially contributed to the current Paraguay gene pool, Figure S9: MDS plot of the pairwise F_ST_ genetic distances between R1b- M269 sub-clades in Paraguay and in European populations that have potentially contributed to the current Paraguay gene pool, Figure S10: Principal component analysis of R1b-L23, R1b-M269, R1b-U106, R1b-S116, R1b-U152 and R1b-M529 haplogroup frequencies in Paraguay, Brazil and in European populations, Figure S11: Frequency distributions on the proportions of European ancestry in admixed South American populations, Table S1: List of genotypes for 46 AIM-InDels obtained in 522 samples from Paraguay, Table S2: List of mtDNA haplotypes and corresponding haplogroups for 417 samples from Paraguay, Table S3: Population data used for mtDNA comparison analyses (F_ST_s) to calculate frequency distributions of the native macrohaplogroups A, B, C and D in South American (Figure 4), and to determine the geographic origin and incidence of haplogroups (Supplementary Figures S4–S7), Table S4: Population data used to search for mtDNA shared haplotypes, Table S5: AMOVA values for Y chromosome, mtDNA and AIM-InDels data, Table S6: Y-SNP haplogroup distribution on eastern Paraguay, Information S1: Y-SNP typing.
