# Supplementary material for: The Ancestry of Eastern Paraguay: A Typical South American Profile with a Unique Pattern of Admixture
Source: Genes (Basel). 2021 Nov 12;12(11):1788. doi: 10.3390/genes12111788 (PMC8625094; doi:10.3390/genes12111788)
Supplement: Supplementary file 1 [file genes-12-01788-s001.zip › supplementary/SUPPLEMENTARY FIGURES_R2.pdf]

SUPPLEMENTARY FIGURES

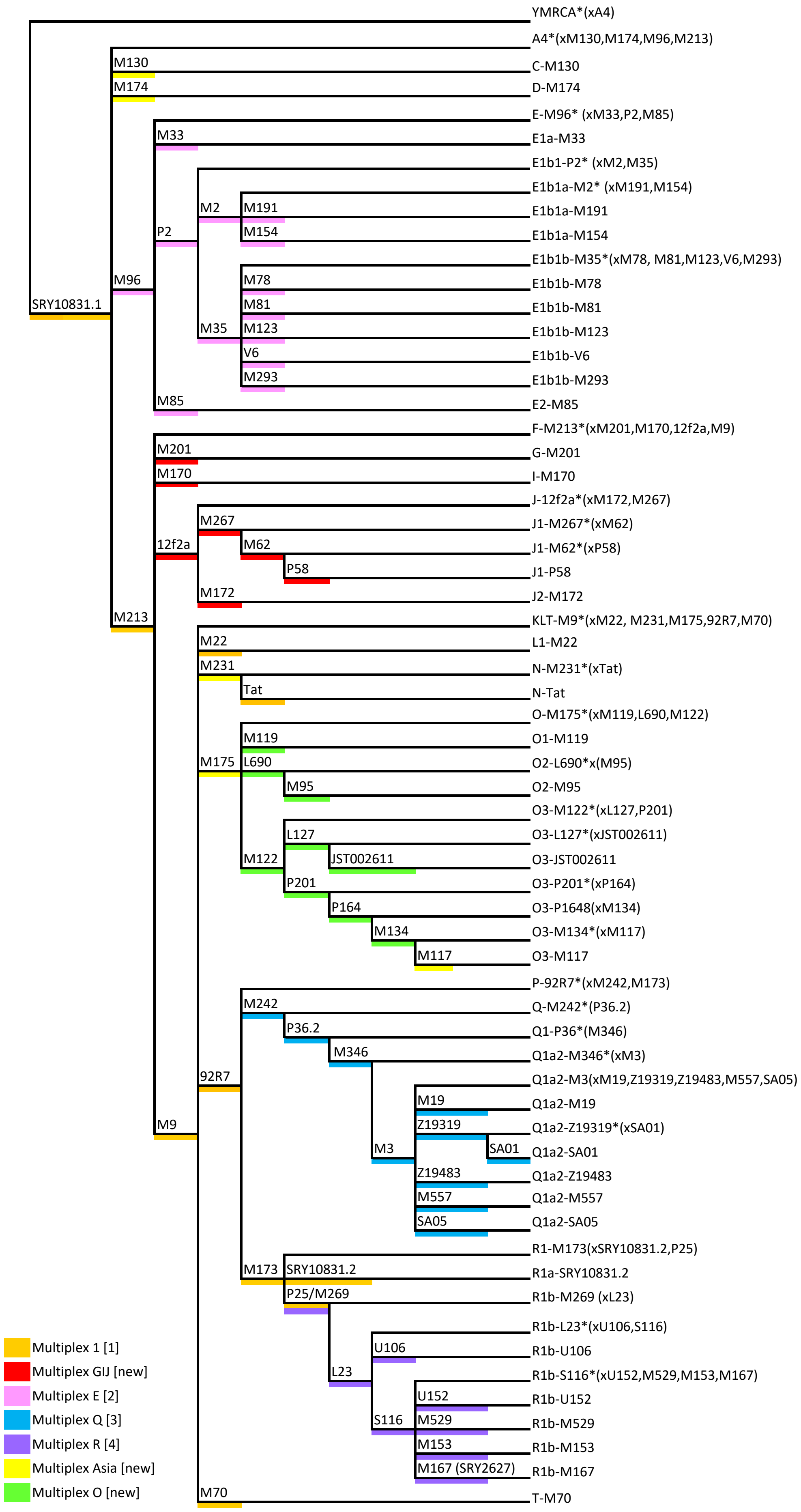

Supplementary Figure S1. Phylogenetic tree of the Y-SNPs analysed in this study. Multiplexes are separated by colour. Haplogroups are named in accordance with van Oven et al. (2014) Hum Mutat 35:187-191. For details on the new multiplexes see Supplementary Information S1. Legend: [1] Brion et al. (2004); [2] Gomes et al. (2010); [3] Aragão (2018); [4] Resque et al. (2016)

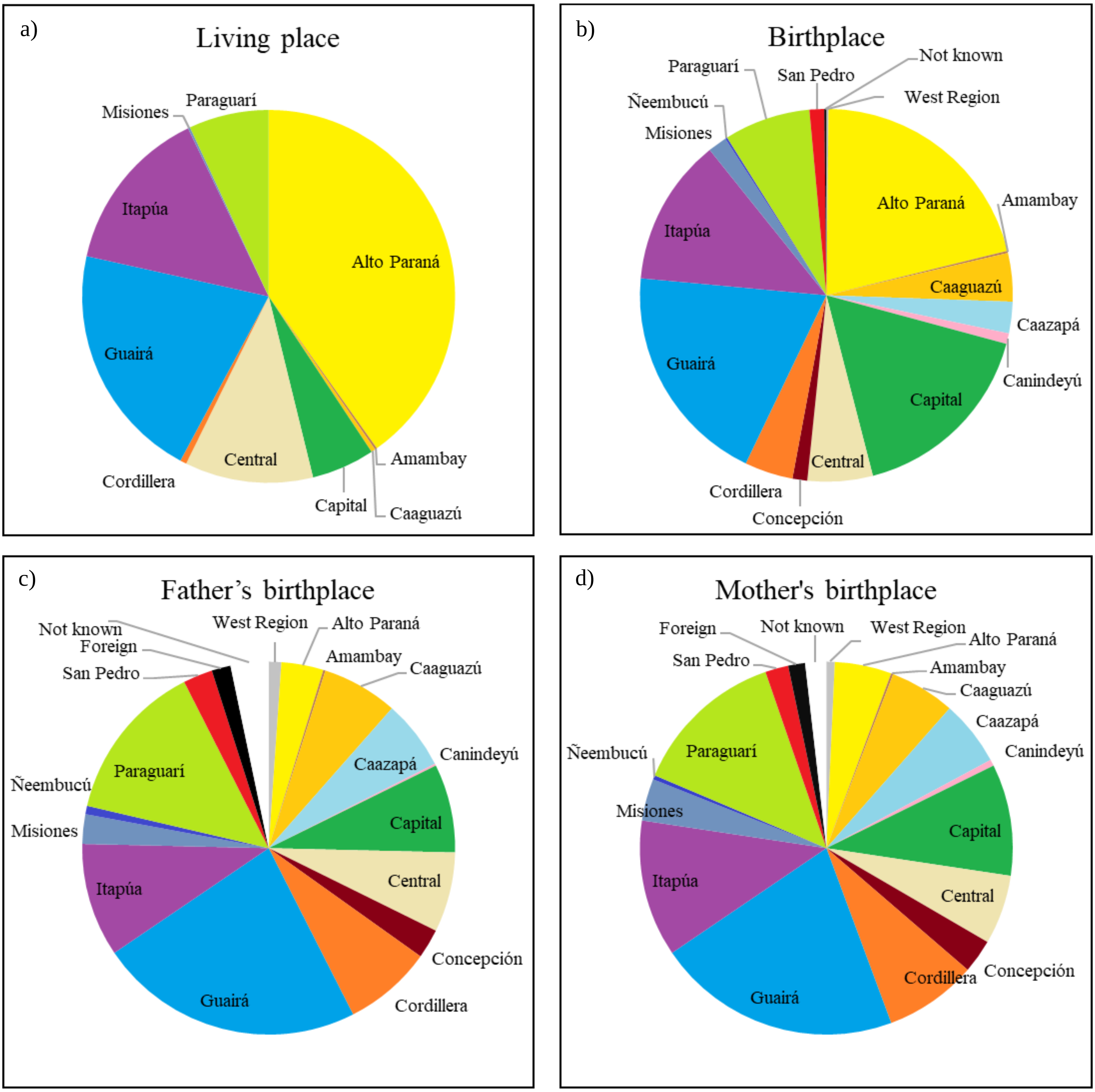

Supplementary Figure S2. Distribution of the samples according to: (a) individual's living place; (b) individual's birthplace; (c) father's birthplace; (d) mother's birthplace.

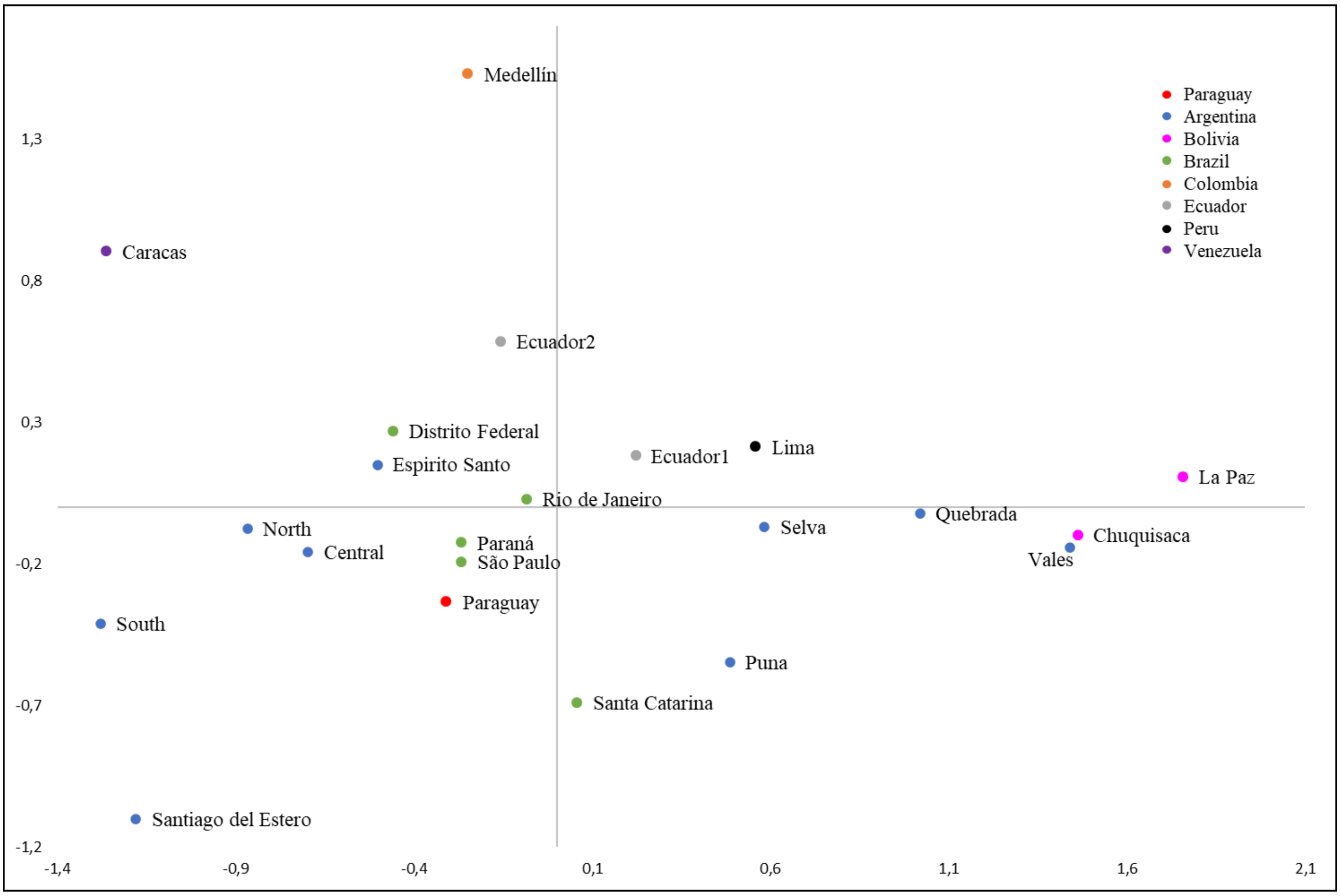

Supplementary Figure S3. Multidimensional scaling (MDS) plot based on F_{ST} genetic distances of native mtDNA haplotype frequencies among admixed populations from Argentina, Bolivia, Brazil, Colombia, Ecuador, Peru and Venezuela. For details on populations used see Supplementary Table S3. Only CR haplotypes were considered. Stress = 0.0769196

Note: Ecuador1- Brandini et al. (2018); Ecuador2 - Baeta et al. (2014)

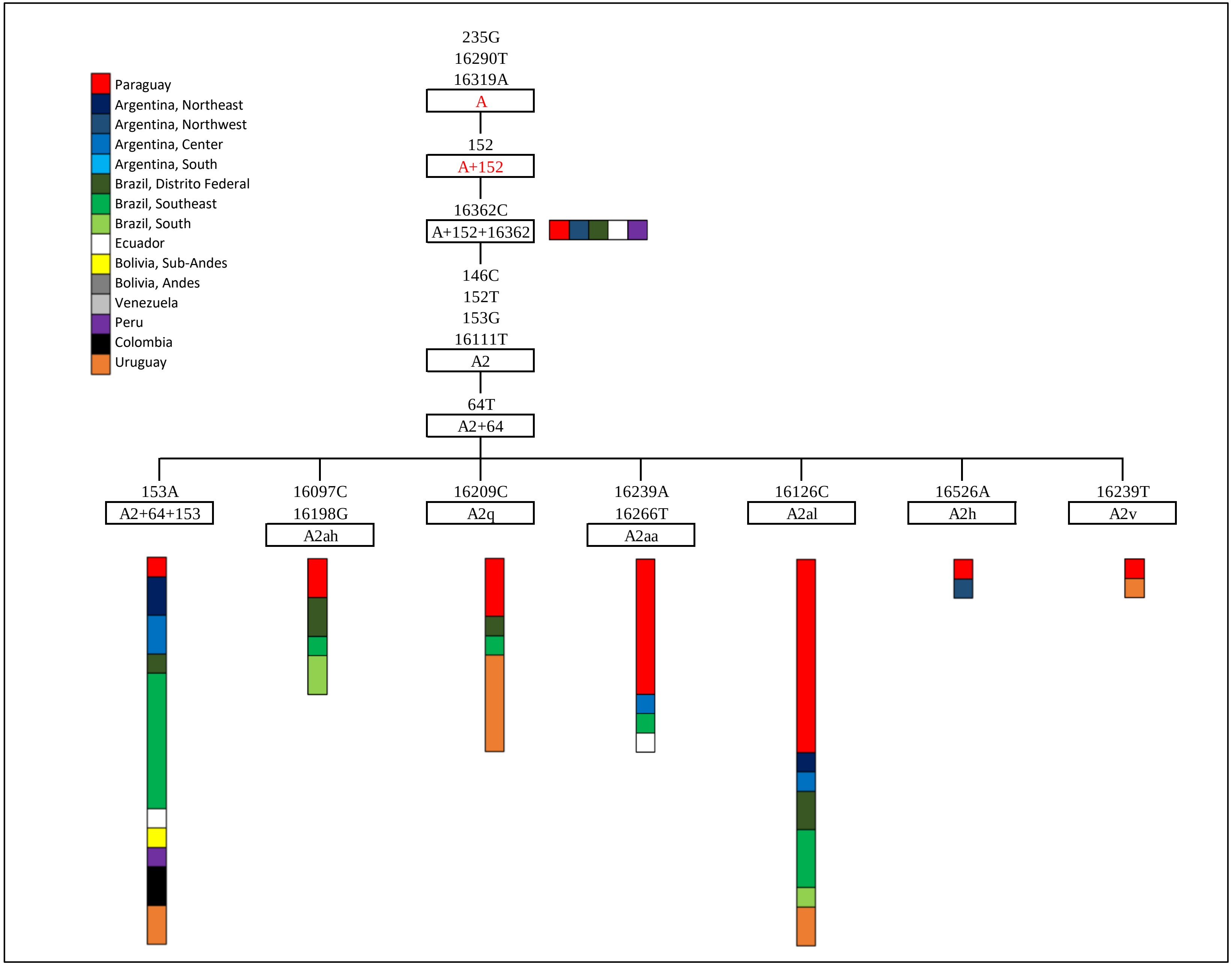

Supplementary Figure S4. Geographic origin and incidence in South America of the A lineages found in Paraguay. Haplogroups in red were not found in Paraguay samples and are only indicated to facilitate interpretation. For details on populations used see Supplementary Table S3. Only control region polymorphisms were considered. The following indel positions were discarded: 16193.xC, 309.xC, 315.xC, 523-524del, 524.xC, 573.xC. The geographic origin and incidence of lineages A2 and A+64 are not depicted due to the high number of samples found for each of these branches.

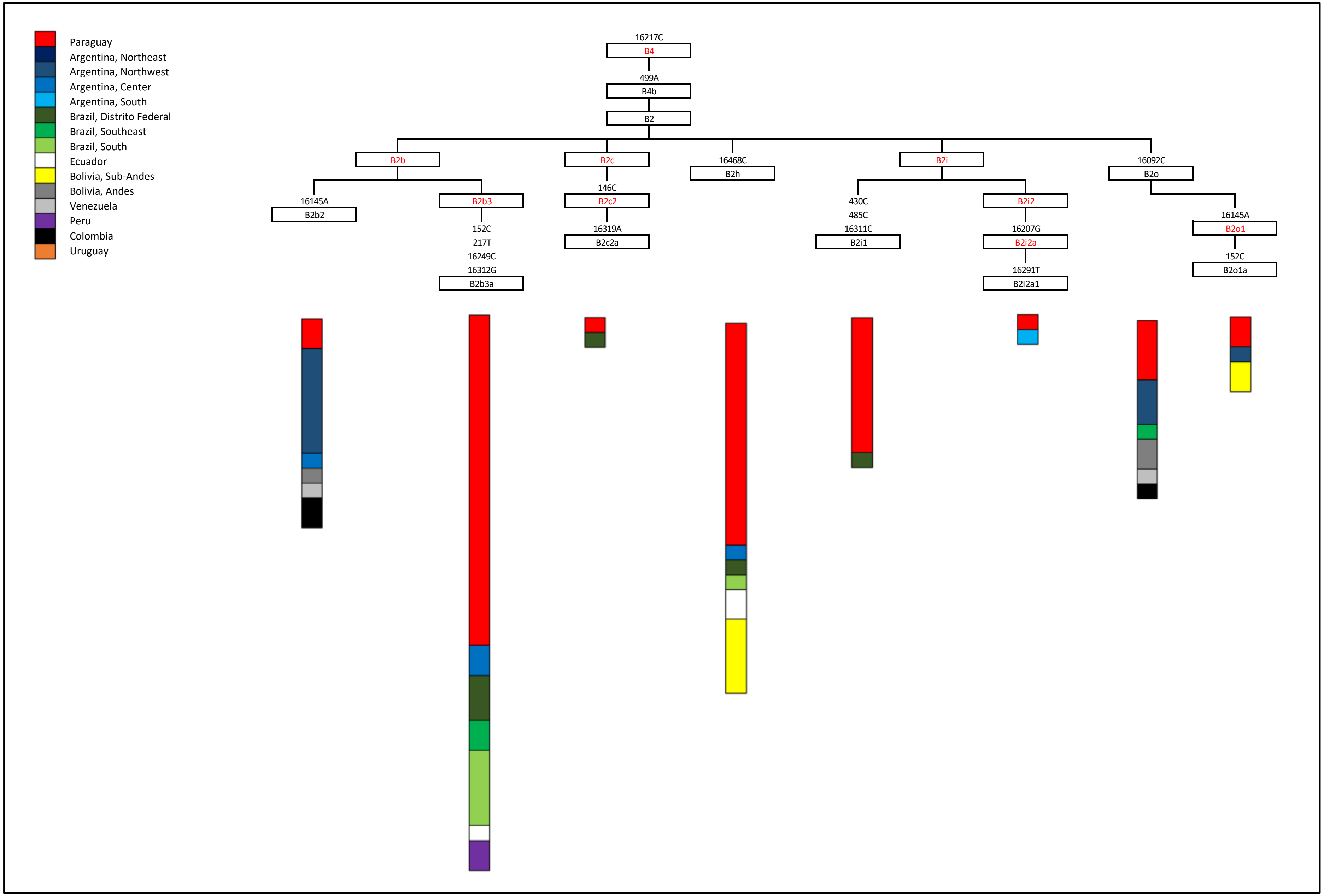

Supplementary Figure S5. Geographic origin and incidence in South America of the B lineages found in Paraguay. Haplogroups in red were not found in Paraguay samples and are only indicated to facilitate interpretation. For details on populations used see Supplementary Table S3. Only control region polymorphisms were considered. The following Indel positions were discarded: 16193.xC, 309.xC, 315.xC, 523-524del, 524.xC, 573.xC. The geographic origin and incidence of lineages B2 and B4b are not depicted due to the high number of samples found for each of these branches.

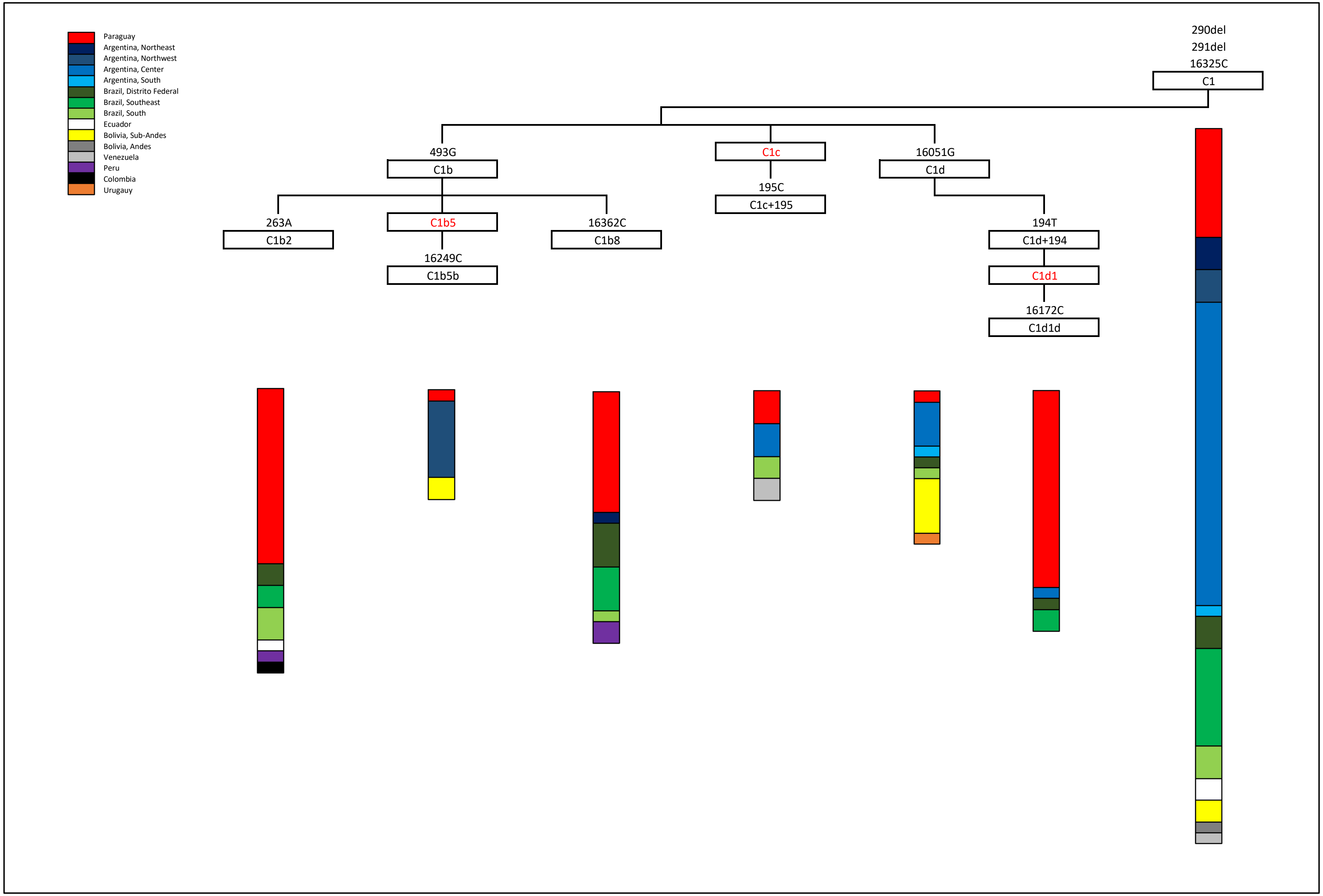

Supplementary Figure S6. Geographic origin and incidence in South America of the C lineages found in Paraguay. Haplogroups in red were not found in Paraguay samples and are only indicated to facilitate interpretation. For details on populations used see Supplementary Table S3. Only control region polymorphisms were considered. The following Indel positions were discarded: 16193.xC, 309.xC, 315.xC, 523-524del, 524.xC, 573.xC. The geographic origin and incidence of lineages C1b and C1d+194 are not depicted due to the high number of samples found for each of these branches.

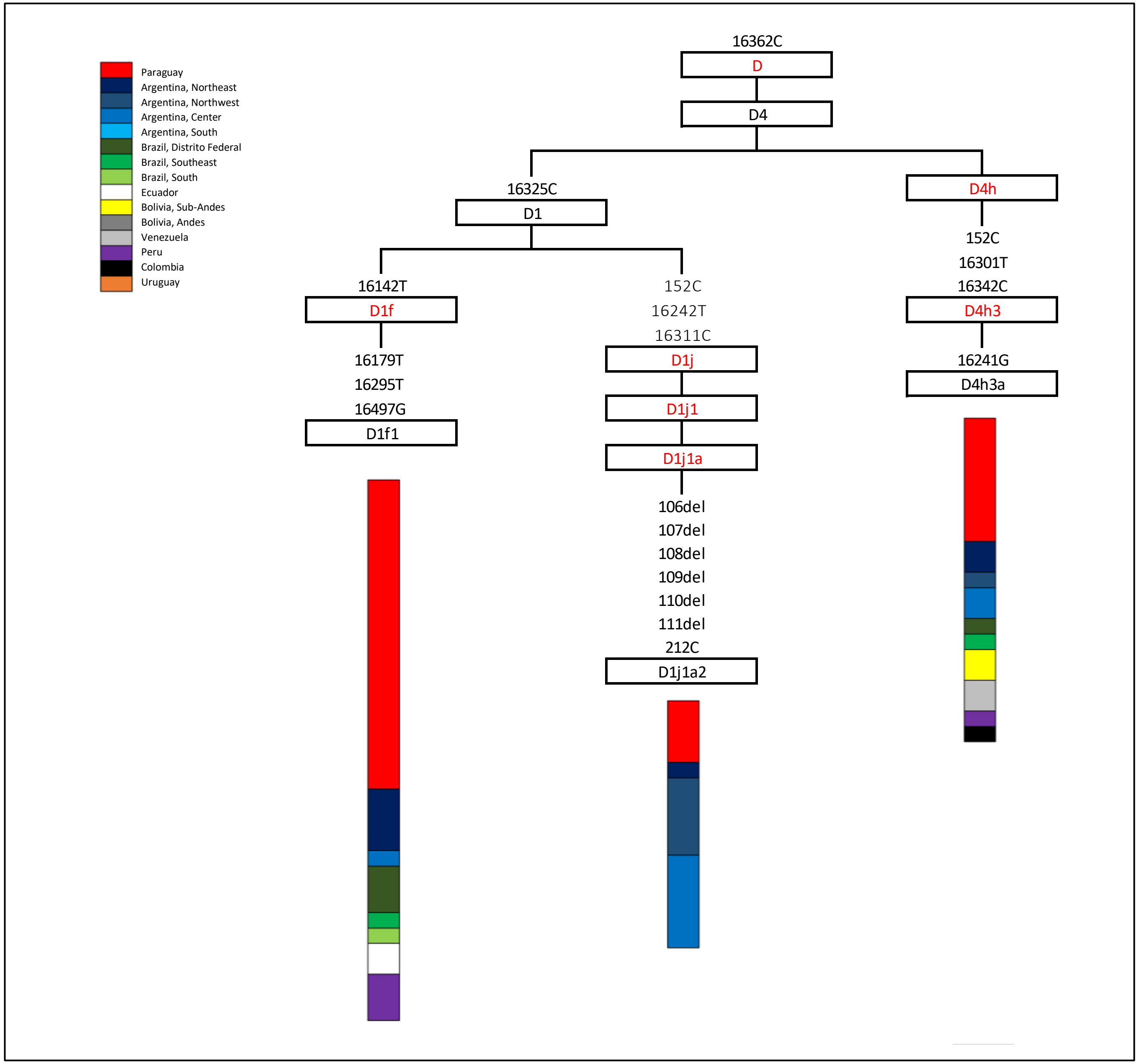

Supplementary Figure S7. Geographic origin and incidence in South America of the D lineages found in Paraguay. Haplogroups in red were not found in Paraguay samples and are only indicated to facilitate interpretation. For details on populations used see Supplementary Table S3. Only control region polymorphisms were considered. The following Indel positions were discarded: 16193.xC, 309.xC, 315.xC, 523-524del, 524.xC, 573.xC. The geographic origin and incidence of lineages D1 and D4 are not depicted due to the high number of samples found for each of these branches.

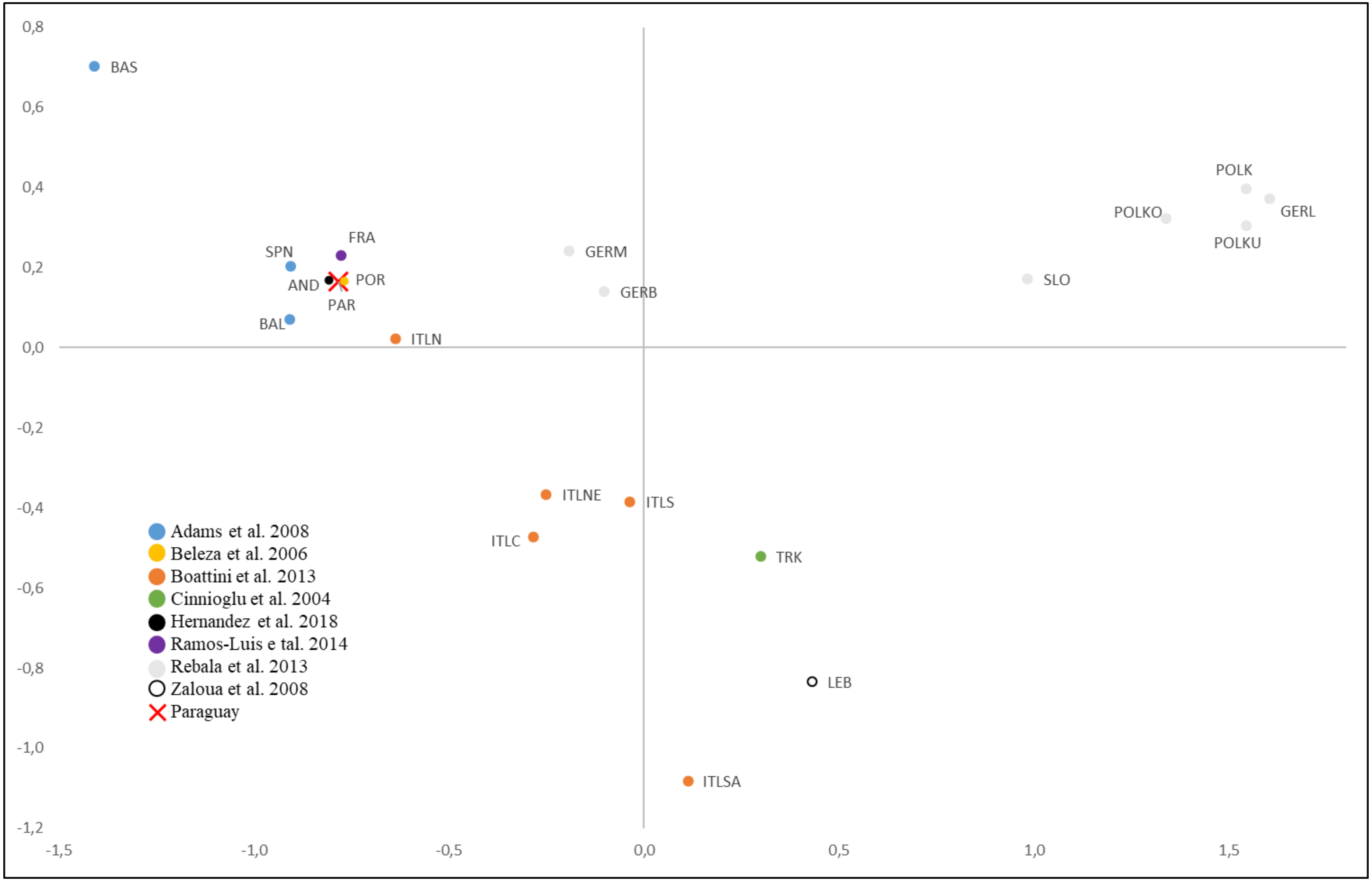

Supplementary Figure S8. MDS plot of the pairwise F_{ST} genetic distances between European Y chromosome haplogroup frequencies in Paraguay and in European populations. Stress = 0.0542318. Legends: AND – Andalusia; BAL – Balearic; BAS – Basque; LEB – Lebanon; FRA – France; GERB – Germany (Bavaria); GERL – Germany (Lusatia); GERM – Germany (Mecklenburg); ITLC – Italy (Central); ITLSA – Italy (Sardinia); ITLS – Italy (South-Eastern); ITLNE – Italy (North-Eastern); ITLN – Italy (North-Western); PAR – Paraguay; POLK – Poland Kaszuby; POLKO – Poland Kociewie; POLKU – Poland Kurpe; POR – Portugal; SLO – Slovakia; SPN – Spain; TRK – Turkey.

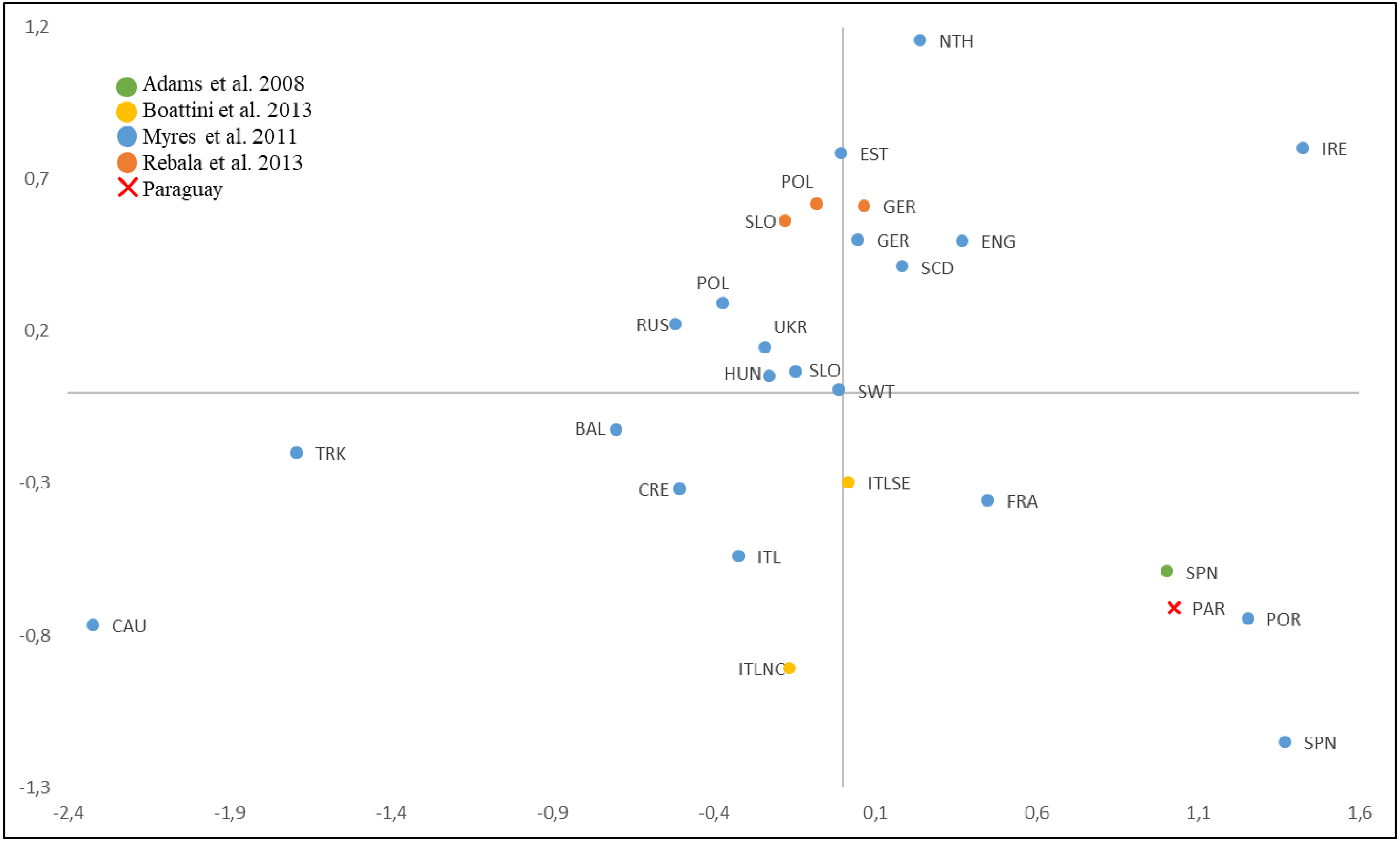

Supplementary Figure S9. MDS plot of the pairwise F_{ST} genetic distances between R1b-M269 sub-clades in Paraguay and in European populations. Legends: BAL – Balkans; CAU – Caucasus; CRE – Crete; ENG – England; EST – Estonia; FRA – France; GER – Germany; HUN – Hungary; IRE –Ireland; ITL – Italy; ITLSE – Italy (South-Eastern); ITLNC – Italy (North/Central); NTH – Netherlands; PAR – Paraguay; POL – Poland; POR – Portugal; RUS – Russia; SCD – Scandinavia; SLO – Slovakia; SPN – Spain; SWT – Switzerland; TRK – Turkey.

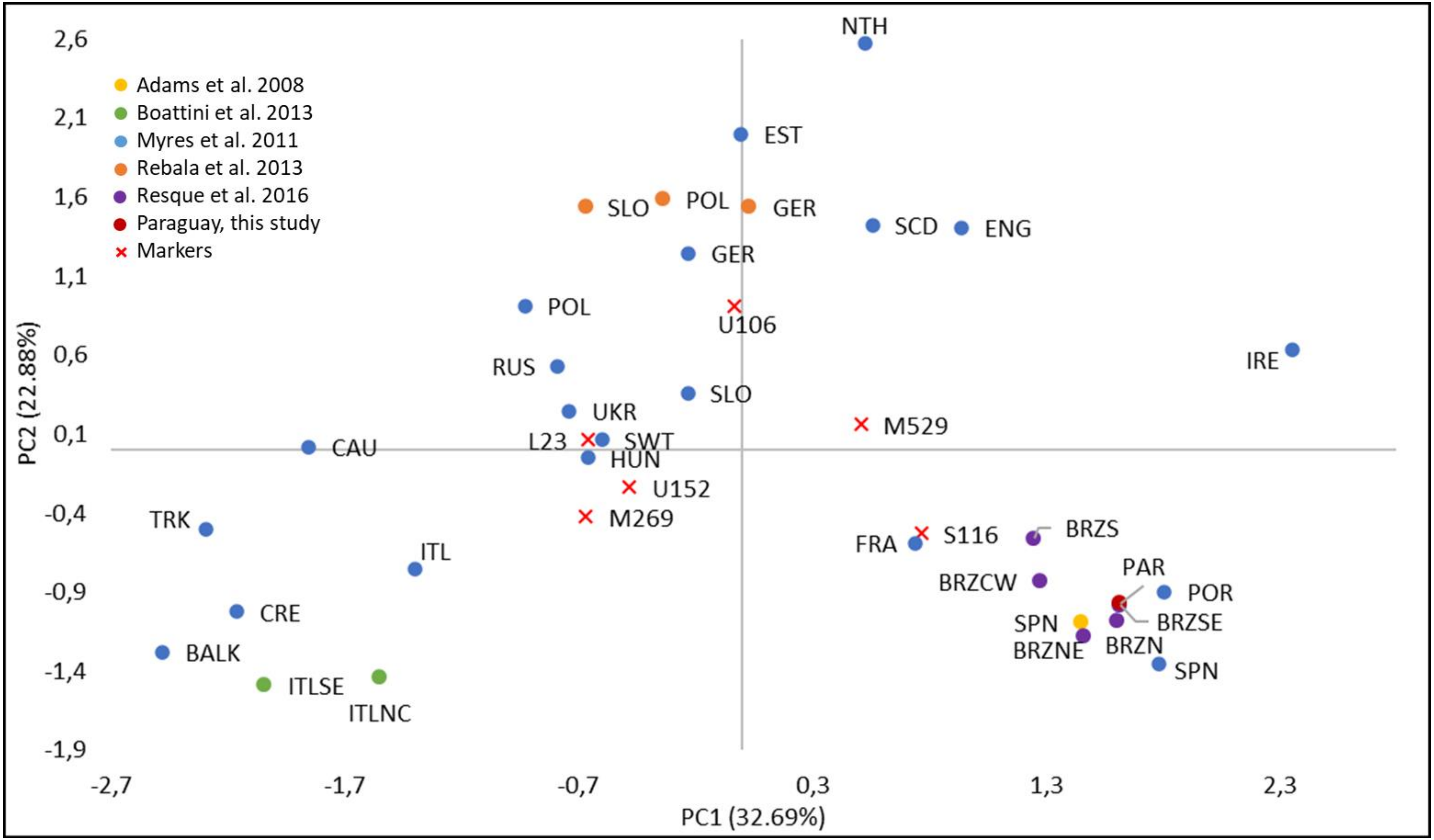

Supplementary Figure S10 - Principal component analysis of R1b-L23, R1b-M269, R1b-U106, R1b-S116, R1b-U152 and R1b-M529 haplogroup frequencies in Paraguay, Brazil and European populations. Legends: BALK – Balkans; BRZCW – Brazil (Central West); BRZN – Brazil (North); BRZNE – Brazil (Northeast); BRZS – Brazil (South); BRZSE – Brazil (Southeast); CAU – Caucasus; CRE – Crete; ENG – England; EST – Estonia; FRA – France; GER – Germany; HUN – Hungary; ITL – Italy; PAR – Paraguay; POL – Poland; POR – Portugal; RUS – Russia; SCD – Scandinavia; SLO – Slovakia; SPN – Spain; SWT – Switzerland; TRK – Turkey.

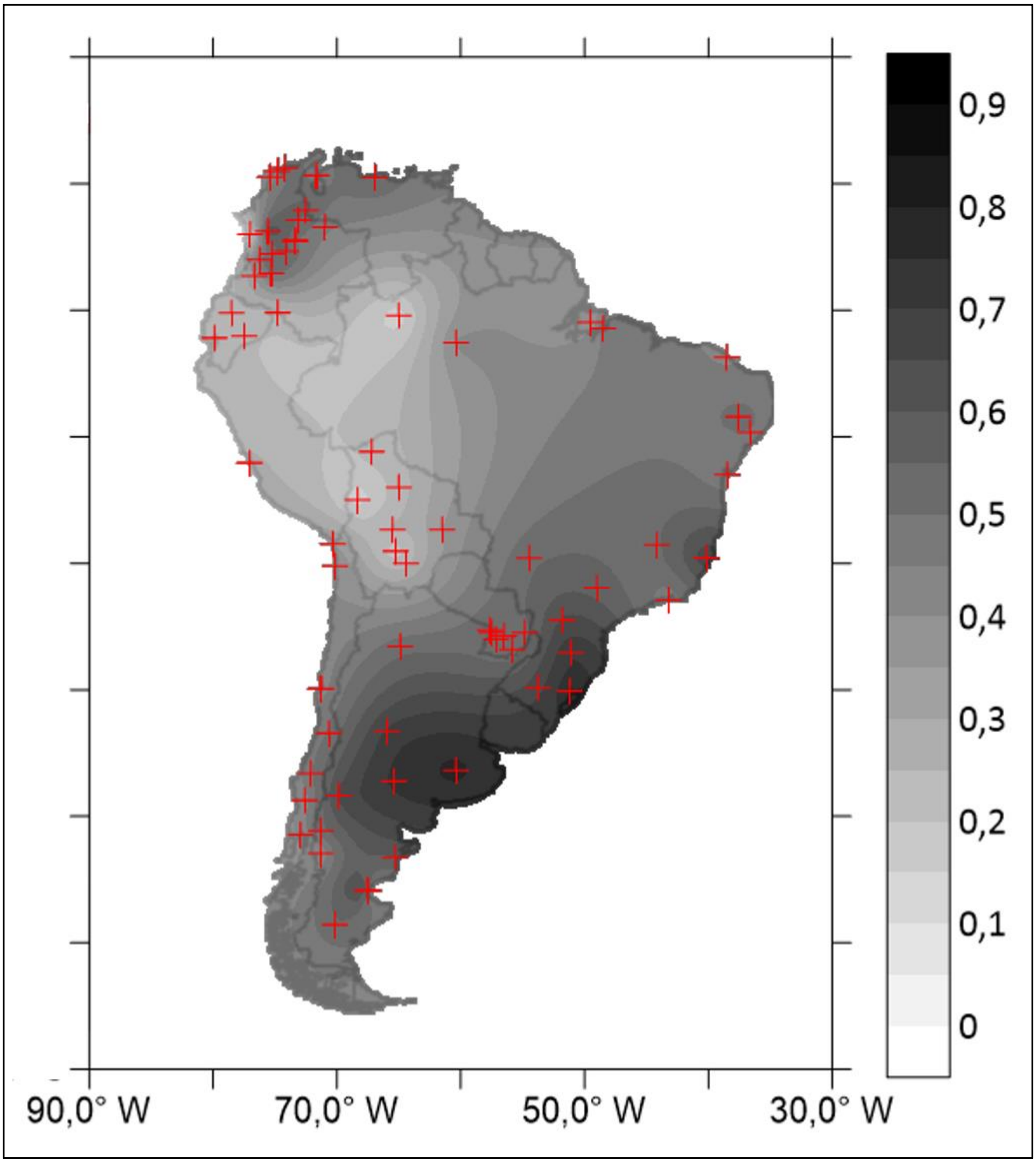

Supplementary Figure S11. Frequency distributions on the proportions of European ancestry in admixed South American populations. Latitude and longitude are represented at the left and bottom sides, respectively. Haplogroup frequencies are represented in grey scale according to the legend in the right side of the box. Red dots represent the location of population used.

References:

Aragão, G. Characterization of male lineages in the Ashaninka from Peru. Master thesis. University of Porto, Porto, Portugal, 2018

Adams, S.M.; Bosch, E.; Balaresque, P.L.; Ballereau, S.J.; Lee, A.C.; Arroyo, E.; López-Parra, A.M.; Aler, M.; Grifo, M.S.G.; Brion, M.; et al. The Genetic Legacy of Religious Diversity and Intolerance: Paternal Lineages of Christians, Jews, and Muslims in the Iberian Peninsula. Am. J. Hum. Genet. 2008, 83, 725–736, doi:10.1016/j.ajhg.2008.11.007.

Baeta, M.; Núñez, C.; Sosa, C.; Bolea, M.; Casalod, Y.; González-Andrade, F.; Roewer, L.; Martínez-Jarreta, B. Mitochondrial diversity in Amerindian Kichwa and Mestizo populations from Ecuador. Int. J. Legal Med. 2012, 126, 299–302, doi:10.1007/s00414-011-0656-4.

Beleza, S.; Gusmão, L.; Lopes, A.; Alves, C.; Gomes, I.; Giouzeli, M.; Calafell, F.; Carracedo, A.; Amorim, A. Micro-Phylogeographic and Demographic History of Portuguese Male Lineages. Ann. Hum. Genet. 2006, 70, 181–194.

Boattini, A.; Martinez-Cruz, B.; Sarno, S.; Harmant, C.; Useli, A.; Sanz, P.; Yang-Yao, D.; Manry, J.; Ciani, G.; Luiselli, D.; et al. Uniparental Markers in Italy Reveal a Sex-Biased Genetic Structure and Different Historical Strata. PLoS One 2013, 8, doi:10.1371/journal.pone.0065441.

Brion, M.; Sobrino, B.; Blanco-Verea, A.; Lareu, M. V.; Carracedo, A. Hierarchical analysis of 30 Y-chromosome SNPs in European populations. Int. J. Legal Med. 2005, 119, 10–15, doi:10.1007/s00414-004-0439-2.

Brandini, S.; Bergamaschi, P.; Fernando Cerna, M.; Gandini, F.; Bastaroli, F.; Bertolini, E.; Cereda, C.; Ferretti, L.; Gómez-Carballa, A.; Battaglia, V.; et al. The Paleo-Indian entry into South America according to mitogenomes. Mol. Biol. Evol. 2018, 35, 299–311, doi:10.1093/molbev/msx267.

Cinnioğlu, C.; King, R.; Kivisild, T.; Kalfoğlu, E.; Atasoy, S.; Cavalleri, G.L.; Lillie, A.S.; Roseman, C.C.; Lin, A.A.; Prince, K.; et al. Excavating Y-chromosome haplotype strata in Anatolia. Hum. Genet. 2004, 114, 127–148, doi:10.1007/s00439-003-1031-4.

Gomes, V.; Sánchez-Diz, P.; Amorim, A.; Carracedo, Á.; Gusmão, L. Digging deeper into East African human Y chromosome lineages. Hum. Genet. 2010, 127, 603–613.

Hernández, C.L.; Dugoujon, J.M.; Sánchez-Martínez, L.J.; Cuesta, P.; Novelletto, A.; Calderón, R. Paternal lineages in southern Iberia provide time frames for gene flow from mainland Europe and the Mediterranean world. Ann. Hum. Biol. 2019, 46, 63–76, doi:10.1080/03014460.2019.1587507.

Myres, N.M.; Rootsi, S.; Lin, A.A.; Järve, M.; King, R.J.; Kutuev, I.; Cabrera, V.M.; Khusnutdinova, E.K.; Pshenichnov, A.; Yunusbayev, B.; et al. A major Y-chromosome haplogroup R1b Holocene era founder effect in Central and Western Europe. Eur. J. Hum. Genet. 2011, 19, 95–101, doi:10.1038/ejhg.2010.146.

Ramos-Luis, E.; Blanco-Verea, A.; Brión, M.; Van Huffel, V.; Sánchez-Diz, P.; Carracedo, A. Y-chromosomal DNA analysis in French male lineages. Forensic Sci. Int. Genet. 2014, 9, 162–168, doi:10.1016/j.fsigen.2013.12.008.

Rębała, K.; Martínez-Cruz, B.; Tönjes, A.; Kovacs, P.; Stumvoll, M.; Lindner, I.; Büttner, A.; Wichmann, H.E.; Siváková, D.; Soták, M.; et al. Contemporary paternal genetic landscape of Polish and German populations: From early medieval Slavic expansion to post-World War II resettlements. Eur. J. Hum. Genet. 2013, 21, 415–422, doi:10.1038/ejhg.2012.190.

Resque, R.; Gusmão, L.; Geppert, M.; Roewer, L.; Palha, T.; Alvarez, L.; Ribeiro-Dos-santos, Â.; Santos, S. Male lineages in Brazil: Intercontinental admixture and stratification of the European background. PLoS One 2016, 11, 1–17, doi:10.1371/journal.pone.0152573.

van Oven M, Van Geystelen A, Kayser M, Decorte R, Larmuseau MH. Seeing the wood for the trees: a minimal reference phylogeny for the human Y chromosome. Hum Mutat. 2014, 35(2):187-191, https://doi.org/10.1002/humu.22468

Zalloua, P.A.; Xue, Y.; Khalife, J.; Makhoul, N.; Debiane, L.; Platt, D.E.; Royyuru, A.K.; Herrera, R.J.; Hernanz, D.F.S.; Blue-Smith, J.; et al. Y-Chromosomal Diversity in Lebanon Is Structured by Recent Historical Events. Am. J. Hum. Genet. 2008, 82, 873–882, doi:10.1016/j.ajhg.2008.01.020.
