# Supplementary material for: The Ancestry of Eastern Paraguay: A Typical South American Profile with a Unique Pattern of Admixture
Source: Genes (Basel). 2021 Nov 12;12(11):1788. doi: 10.3390/genes12111788 (PMC8625094; doi:10.3390/genes12111788)
Supplement: Supplementary file 1 [file genes-12-01788-s001.zip › supplementary/SUPPLEMENTARY INFORMATION.pdf]

SUPPLEMENTARY INFORMATION

Y-SNP typing

Y-SNP typing was directed according to haplogroup predictions obtained with the Y-DNA Haplogroup Predictor NevGen tool (http://www.nevgen.org), based on the Y-STR profiles. The Y-SNPs were grouped hierarchically into seven multiplexes. The Y-SNPs typed in this study are listed in the Supplementary Figure S2, as well as the PCR/SNaPshot multiplex strategies followed. The multiplexes 1 (Brión *et al*. 2004), E (Gomes *et al*. 2010), Q (Aragão, 2018) and R (Resque *et al*. 2016) were previously described in the literature. The multiplexes GIJ, Asian and O are described below.

Multiplexes design

To detect GIJ branches in Paraguay, 7 Y-SNPs were included in a single multiplex reaction, namely M201, M170, 12f2.a, M267, M62, P58 and M172 (Supplementary Fig. S2).
Few samples were detected outside the main European, African, and Native American haplogroups. Two multiplexes were therefore optimized to detect the basal Asian haplogroups C, D, N and O (Multiplex Asia), as well as sub-lineages inside haplogroup O (Multiplex O). These multiplexes included 14 SNPs: M175, M174, M130, M231, M117, M119, L690, M95, M122, L127, JST002611, P201, P16 and M134 (Supplementary Fig. S2).
Sequences encompassing 500 bps flanking the SNPs were retrieved from the GenBank. The software Primer3web version 4.1.0 (Untergasser *et al*. 2012) was used to design primer pairs. The final primers were selected based on the melting temperature and the predicted amplicon sizes (Table SI1). Single-based extension (SBE) primers were also design as previously described, under the extra requirement that the SBE primers 3'-end had to be complementary to the base immediately before the selected SNP. To enable size-based separation of Y-SNPs by electrophoresis, polynucleotide tails of different lengths were added to the primers. For primers included in the same multiplex reaction, primer-dimer and hairpin structures were screened using the AutoDimer tool (Vallone and Butler, 2004). See primer sequences in Table SI1.

PCR amplification and Single base sequencing

All PCR multiplexes were performed using 2 µM of each primer, 2-5 ng of DNA and 2.5 µl QIAGEN Multiplex PCR Master Mix (Qiagen), in a total volume of 5 µl. PCR conditions were standardized to an initial denaturation at 96ºC for 15 minutes, followed by 35 cycles at 95ºC for 30 seconds, 60ºC for 60 seconds and 72ºC for 90 seconds. Amplification products were purified using ExoSAP_IT™ (Applied Biosystems), with the following settings: enzyme activation at 37ºC for 15 minutes, and inactivation at 85ºC for 15 minutes. Single base extension reactions were performed in 5 µl final volume, with 1 µl of SNaPshot® Multiplex Kit (Applied Biosystems), 1 µl of the SBE mix (see SBE primer concentrations in Table SI1) and 1.5 µl of the purified PCR product. Thermocycling conditions were: 96ºC for 10 seconds, 50ºC for 5 seconds and 60ºC for 30 seconds, for 25 cycles. The final purification was performed with SAP (Applied Biosystems). One unit of SAP was mixed with 5 µl of SNaPshot product and incubated at 37ºC for 60 minutes and 85ºC for 15 minutes. Fragment separation and detection were accomplished in a ABI3500 (Applied Biosystems), using GeneScan™ 120 LIZ™ as internal size standard (Applied Biosystems). The results were analysed with the GeneMapper® v.4.0 software.

Table SI1. List of primers used for multiplex design. Polynucleotide tails added to the primers are indicated in lowercase.

	Marker	SNP	PCR Forward Primer (5'-3')	PCR Reverse Primer (5'-3')	PCR fragment size (bp)	SBE Primer (5'-3')	[ ] in SNaPshot reaction (µM)
Multiplex GIJ	M62	T/C	ACTAAAACACCATTAGAAACAAAGG	CTGAGCAACATAGTGACCCC	309	ctgcaaCAATGTTTGTTGGCCATGGA	1.2
	M267	T/G	CGTTGTCCCTGTGTTTCCAT	CTGTTGCCCAGGCTAGTGTC	256	tgaaagtctgacaaCTTCCACACAAAATACTGAAMGT	1.2
	M172	T/G	TCCTCATTCACCTGCCTCTC	TCCATGTTGGTTTGGAACAG	187	AAACCCATTTTGATGCTT	0.8
	P58	T/C	ACAGGAGGCCATAATGCAAC	GAGCCTCACACCTTCCTCTG	180	cacgtcgtgaaagtctgacaaTGACATTTGTGTGCTTTGC	1.2
	M170	A/C	TGCAGCTCTTATTAAGTTATGTTTTCA	CCAATTACTTTCAACATTTAAGACC	158	ACACAACCCACACTGAAAAAAA	0.8
	M201	G/T	TCAAATTGTGACACTGCAATAGTT	CATCCAACACTAAGTACCTATTACGAA	144	aagtctgacaaTAATAATCCAGTATCAACTGAGG	0.1
	12f2	del Alu	CACTGACTGATCAAAATGCTTACAGAT	GGATCCCTTCCTTACACCTTATACA	90	*Genotyped by fragment size*	-
Multiplex Asia	M231	G/A	TTGCCTGTGCATGAAAAACT	TTTGACACCACAGAAATTACAGG	277	ACAACATTTACTGTTTCTACTGCTTTC	0.3
	M117	del AGAT	ATTTGGGTAGAAAAACTGCAAG	ACCAAAGGAATGCACATCTATCT	219	*Genotyped by PCR allele specific*	-
	M174	T/C	TCTCCGTCACAGCAAAAATG	GAAGGTCCTGGAGATGCAAA	200	CACCCCTCACTTCTGCACT	0.2
	M175	del TTCTC	gtctgacaaACATGCCTTCTCACTTCTCTTCTC	TCTTGCAGCATTTTCAGTTAGC	183	*Genotyped by PCR allele specific*	-
	M130	C/T	TGAGGCATGTGTGTTTTGGT	TGGCCAGCCTCTTATCTCTC	167	GGGCAATAAACCTTGGATTTC	0.4
Multiplex O	M134	del C	CCCTTCTTTGGCTTCTCTTTG	TTTCCCCACAACCAGACAAT	260	agtctgacaaAAGAAAAGGCCCAGGAAAGTAT	0.4
	L127	G/A	CCCTAAAACAAGGGGTCGAT	GGGGAAGGGATAGCATTAGG	259	cgtcgtgaaagtctgacaaTCAGAATAATAACAGAGTAATTGGCAG	0.5
	JST002611	C/T	AGCCAACATACTCGCCAATC	GGCTTGCCCTACTGAGAACA	257	ccacgtcgtgaaagtctgacaaCGAGGCCCTGTGCTTCCAGA	0.4
	M122	T/C	GGTATTCAGGCGATGCTGAT	GTGACTGCAAATGGTATGCAA	220	gacaaTCAGATTTTCCCCTGAGAGC	0.36
	M95	C/T	CCTTCTTGGGATCAAATGGA	GTTGTGAGGTCCTTCCCAGA	219	GGATAAGGAAAGACTACCATATTAGTG	0.4
	L690	A/G	CTCTCTCATGGGCTGGACAT	TGGCAGATGCAAGCTACCTA	207	ctgacaaAGGCAGGTATTCAGAGAAGAAGCAA	0.36
	P201	T/C	TGTGCTGTGCAAGTTGTGTG	AACCCCAAATCCCAAGGTAG	152	gtctgacaaGTGAGAGCCAGTTAAAGCCC	0.24
	M119	A/C	TGGGTTATTCCAATTCAGCA	CAAACCGCAGTGCTATGTGT	95	gtgaaagtctgacaaTTATTCCAATTCAGCATACAGGC	0.4
	P164	T/C	AGCATTTTGGTCCCATCTTTT	CCCTCTTTTTCCTCCCATTC	78	AGCATTTTGGTCCCATCTTTT	0.3

References

Aragão, G. Characterization of male lineages in the Asháninka from Peru. Master thesis, University of Porto, Porto, Portugal, **2018.**

Brión, M.; Sobrino, B.; Blanco-Verea, A.; Lareu, M. V.; Carracedo, A. Hierarchical analysis of 30 Y-chromosome SNPs in European populations. Int. J. Legal Med. **2005**, 119, 10–15, doi:10.1007/s00414-004-0439-2.

Gomes, V.; Sánchez-Diz, P.; Amorim, A.; Carracedo, Á.; Gusmão, L. Digging deeper into East African human Y chromosome lineages. Hum. Genet. **2010**, 127, 603–613.

Resque, R.; Gusmão, L.; Geppert, M.; Roewer, L.; Palha, T.; Alvarez, L.; Ribeiro-Dos-santos, Â.; Santos, S. Male lineages in Brazil: Intercontinental admixture and stratification of the European background. PLoS One **2016**, 11, 1–17, doi:10.1371/journal.pone.0152573.

Untergasser, A.; Cutcutache, I.; Koressaar, T.; Ye, J.; Faircloth, B.C.; Remm, M.; Rozen, S.G. Primer3-new capabilities and interfaces. Nucleic Acids Res. **2012**, 40, 1–12, doi:10.1093/nar/gks596.

Vallone, P.M.; Butler, J.M. AutoDimer: A screening tool for primer-dimer and hairpin structures. Biotechniques **2004**, 37, 226–231, doi:10.2144/04372st03.
